# MAPK signaling determines lysophosphatidic acid (LPA)-induced inflammation in microglia

**DOI:** 10.1186/s12974-020-01809-1

**Published:** 2020-04-23

**Authors:** Ioanna Plastira, Eva Bernhart, Lisha Joshi, Chintan N. Koyani, Heimo Strohmaier, Helga Reicher, Ernst Malle, Wolfgang Sattler

**Affiliations:** 1 Division of Molecular Biology and Biochemistry, Gottfried Schatz Research Center, Medical University of Graz, Neue Stiftingtalstrasse 6/6, 8010 Graz, Austria; 2 Division of Cardiology, Department of Internal Medicine, Medical University of Graz, Graz, Austria; 3 Center for Medical Research, Medical University of Graz, Graz, Austria; 4 Center for Explorative Lipidomics, BioTechMed, Graz, Austria

**Keywords:** Bioactive lipids, JNK, p38, ERK1/2, Chemokines, Cytokines, Lipopolysaccharide, Neuroinflammation

## Abstract

**Background:**

In the extracellular environment, lysophosphatidic acid (LPA) species are generated via autotaxin (ATX)-mediated hydrolysis of lysophospholipid precursors. Members of the LPA family are potent lipid mediators transmitting signals via six different G protein-coupled LPA receptors (LPAR1-6). The LPA signaling axis is indispensable for brain development and function of the nervous system; however, during damage of the central nervous system, LPA levels can increase and aberrant signaling events counteract brain function. Here, we investigated regulation of the ATX/LPA/LPAR axis in response to lipopolysaccharide-induced systemic inflammation in mice and potential neurotoxic polarization programs in LPA-activated primary murine microglia.

**Methods:**

In vivo, LPAR1-6 expression was established by qPCR in whole murine brain homogenates and in FACS-sorted microglia. ELISAs were used to quantitate LPA concentrations in the brain and cyto-/chemokine secretion from primary microglia in vitro. Transcription factor phosphorylation was analyzed by immunoblotting, and plasma membrane markers were analyzed by flow cytometry. We used MAPK inhibitors to study signal integration by the JNK, p38, and ERK1/2 branches in response to LPA-mediated activation of primary microglia.

**Results:**

Under acute and chronic inflammatory conditions, we observed a significant increase in LPA concentrations and differential regulation of LPAR, ATX (encoded by ENPP2), and cytosolic phospholipase A2 (encoded by PLA2G4A) gene expression in the brain and FACS-sorted microglia. During pathway analyses in vitro, the use of specific MAPK antagonists (SP600125, SB203580, and PD98059) revealed that JNK and p38 inhibition most efficiently attenuated LPA-induced phosphorylation of proinflammatory transcription factors (STAT1 and -3, p65, and c-Jun) and secretion of IL-6 and TNFα. All three inhibitors decreased LPA-mediated secretion of IL-1β, CXCL10, CXCL2, and CCL5. The plasma membrane marker CD40 was solely inhibited by SP600125 while all three inhibitors affected expression of CD86 and CD206. All MAPK antagonists reduced intracellular COX-2 and Arg1 as well as ROS and NO formation, and neurotoxicity of microglia-conditioned media.

**Conclusion:**

In the present study, we show that systemic inflammation induces aberrant ATX/LPA/LPAR homeostasis in the murine brain. LPA-mediated polarization of primary microglia via MAPK-dependent pathways induces features reminiscent of a neurotoxic phenotype.

## Introduction

Microglia are the predominant type of immune cells in the central nervous system (CNS) that comprise approximately 5–12% of glial cells [1, 2]. Microglia derive from mesodermal progenitors that arise from the yolk sac and colonize the neuroepithelium by day E9.5 [3]. Microglia are a self-renewing, distinct population of tissue macrophages and play fundamental roles in development and homeostasis of the CNS [4]. In the last decade, there has been a tremendous progress in understanding different aspects of microglia biology and this helped to contextualize and analyze their responses under pathological conditions [5].

Neurodegenerative disorders are characterized by one or more disease-specific mechanism(s) that induce inflammatory responses [6]. Activation of the innate immune cells of the CNS is one of the common components shared by these diseases. During the inflammatory process, microglia appear to be heterogeneous with diverse functional phenotypes, ranging from a pro-inflammatory to immunosuppressive/anti-inflammatory phenotype [7]. They can exacerbate disease or promote neuronal survival depending on their activation profile [8]. The recent application of powerful methodologies, such as transcriptomic and proteomic analysis of microglia at a single-cell level revealed unique phenotypic signatures under both physiological and neurodegenerative settings [9–11].

Lysophosphatidic acid (LPA) is a bioactive phospholipid (PL), ubiquitously present in all examined tissues. It acts as an extracellular signaling molecule through six cognate G protein-coupled receptors (GPCRs) [12]. There is a range of structurally related LPA species present in various biological systems [13]. An important aspect of LPA receptor (LPAR) biology is that different LPA species may activate different receptor isoforms [14]. LPA can be synthesized from PLs via phosphatidylserine-specific phospholipase A1 (PS-PLA1), secretory and cytosolic phospholipase A2 (sPLA2 and cPLA2), and autotaxin (ATX), a secreted enzyme with lysophospholipase D activity catalyzing hydrolysis of lysophosphatidylcholine to LPA [12]. In a second pathway, phosphatidic acid (PA), produced from PLs through phospholipase D activity or from diacylglycerol (DAG) through diacylglycerol kinase (DGK) activity, can be converted to LPA [15]. Currently LPAR1-6, which activate heterotrimeric G proteins to initiate various signaling cascades, are characterized. LPARs are expressed in varying spatiotemporal patterns from fetal through mature life, and LPA signaling drives diverse physiological and pathophysiological processes [12].

In the CNS, LPA is found in the embryonic brain, choroid plexus, meninges, neural tube, blood vessels, spinal cord, and cerebrospinal fluid (CSF) at low nanomolar to micromolar concentrations [16]. LPAR expression and LPA-dependent signaling cascades impact microglia functions including proliferation, cell membrane hyperpolarization, enhanced chemokinesis, membrane ruffling, and growth factor upregulation [17–19]. In addition, LPA controls microglial activation and energy homeostasis [20], modulates the oxidative stress response [21], regulates the induction of chronic pain [22], and interferes with pro-inflammatory cytokine production [23].

Beyond development and normal function of the nervous system, LPAR signaling is also important during CNS stress or damage. Following injury, LPA concentrations in the brain and CSF are significantly elevated, and LPAR1-3 expression is upregulated [24]. Moreover, high ATX levels are reported in white matter adjacent to injury lesions in the rat cortex [25], suggesting increased LPA signaling activity. Aberrant LPA signaling contributes to a range of diseases, including neuropathic pain, neurodevelopmental, neuropsychiatric and neurodegenerative disorders, cardiovascular disease, bone disorders, fibrosis, cancer, infertility, and obesity [12].

We reported that LPAR5 transmits pro-inflammatory signals and impacts microglia biology [26]. We identified the LPAR5-protein kinase D (PKD) axis as one possible pathway via which LPA induces an inflammatory and migratory response in both BV-2 and primary murine microglia cells [27]. However, data regarding LPAR signaling during endotoxemia and/or neuroinflammation are scarce. Here we show that endotoxemia increases LPA concentrations and induces differential expression of ENPP2 (encoding ATX) and LPARs in mouse brain and FACS-sorted microglia. In vitro, we analyzed the role of mitogen-activated protein kinase (MAPK) signaling pathways on LPA-induced inflammatory responses of primary murine microglia. We show that LPA-induced downstream signaling events that induce an inflammatory microglia phenotype are mainly executed via MAPK-dependent pathways.

## Materials and methods

### Materials

Cell culture medium RPMI1640 and DMEM (Dulbecco’s modified Eagle’s medium), fetal calf serum (FCS), Hank’s-buffered salt solution (HBSS), DNase I, antibiotics, and trypsin were from Invitrogen (Waltham, MA, USA). LPA (1-oleoyl-2-hydroxy-sn-glycero-3-phosphate; LPA18:1) and 3-(4,5-dimethyl-2-thiazolyl)-2,5-diphenyltetrazolium bromide (MTT) was from Sigma-Aldrich (St. Louis, MO, USA). The pharmacological LPAR5 antagonist TCLPA5 ([5-(3-Chloro-4-cyclohexylphenyl)-1-(3-methoxyphenyl)-1H-pyrazole-3-carboxylic acid]), the c-jun N-terminal kinase 1 (JNK) inhibitor-SP600125, the p38 inhibitor-SB203580, and the MAPK kinase (MEK) inhibitor-PD98059 were from Tocris (Bristol, UK). Phycoerythrin (PE)-CD40, Allophycocyanin (APC)-CD86, and PE-CD206 antibodies and their isotype controls were from Biolegend (San Diego, CA, USA). Antibodies against cyclooxygenase-2 (COX-2), Arginase-1 (Arg1), and the total and phosphorylated forms of ERK1/2, p38 MAPK, JNK, p65-NFkB, cJun, signal transducer and activator of transcription 1 (STAT1), and STAT3 were from Cell Signaling (Beverly, MA, USA). Monoclonal anti-mouse β-actin (clone AC-74) and β-tubulin antibodies and lipopolysaccharide (LPS) from *Escherichia coli* O111:B4 (LPS) were from Sigma-Aldrich (St. Louis, MO, USA).

### Animals

All mice used for the current study were of C57BL/6 J genetic background and group housed on a 12 h/12 h light/dark cycle with food and water ad libitum. The Austrian Federal Ministry of Education, Science and Research, Division of Genetic Engineering and Animal Experiments approved animal experiments (BMWF-66.010/0067-V/3b/2018). All efforts were made to ensure minimum suffering.

### Primary microglia culture

Primary murine microglia (PMM) were isolated from C57BL/6 J cortices of neonatal (P0-P4) mice as previously described [26]. Briefly, the brain cortices were dissected from the whole brain, stripped from their meninges, and minced with scissors into small pieces. Glial cells were separated by trypsinization (0.1% trypsin, 20 min, 37 °C, 5% CO_2_), and the cell suspension was cultured in 75 cm^2^ tissue culture flasks precoated with 5 μg/ml poly-d-lysine (PDL) in DMEM containing 15% FCS, 1% penicillin, 1% streptomycin, and 5 ml l-glutamine (stock 200 mM). After 2 days in culture, the medium was changed to fresh DMEM medium and cells were cultured for another 10 to 14 days. Microglia were removed from the mixed glia cell cultures by smacking the culture flasks 10–20 times and seeded onto PDL-coated cell culture plates for further use.

### BV-2 microglia culture

The murine microglial cell line BV-2 was from Banca Biologica e Cell Factory (Genova, Italy). Cells were cultivated and maintained in RPMI1640 medium supplemented with 10% FCS, 1% penicillin, 1% streptomycin, and 5 ml l-glutamine (stock 200 mM) at 37 °C in a humidified incubator under 5% CO_2_ and 95% air. The culture medium was changed to fresh medium every 2 or 3 days. When cells reached confluency, they were split into new flasks or processed for experiments.

### CATH.a neurons culture

The murine neuronal cell line CATH.a was from ATCC (CRL-11179) and maintained in RPMI1640 medium supplemented with 10% horse serum, 5% FCS, 1% penicillin-streptomycin, 0.4 % HEPES, and 0.2% sodium pyruvate at 37 °C in a humified incubator (5% CO_2_ and 95% air). When cells reached confluency, they were split into new flasks (subcultivation ratio of 1:4) using 0.12% trypsin without EDTA or used immediately for the experiments.

### LPA treatment

Cells were plated in 12- or 24-well PDL-coated plates and allowed to adhere for 2–3 days. Cells were always incubated in serum-free DMEM medium overnight before starting LPA (1 μM) or LPA/inhibitor (added simultaneously) treatments. Aqueous LPA stock solutions (5 mM) were stored at − 80 °C. Only freshly thawed stocks were used for the experiments.

### Treatments with pharmacological inhibitors

The JNK inhibitor SP600125, the p38 inhibitor SB203580, and the MEK inhibitor PD98059 were used in this study. All inhibitors were diluted in DMSO (stock concentrations 10 and 20 mM) and kept at − 20 °C. During the experiments, they were used at a final concentration of 10 μM.

### Intraperitoneal LPS injections

**Fig. 1:**
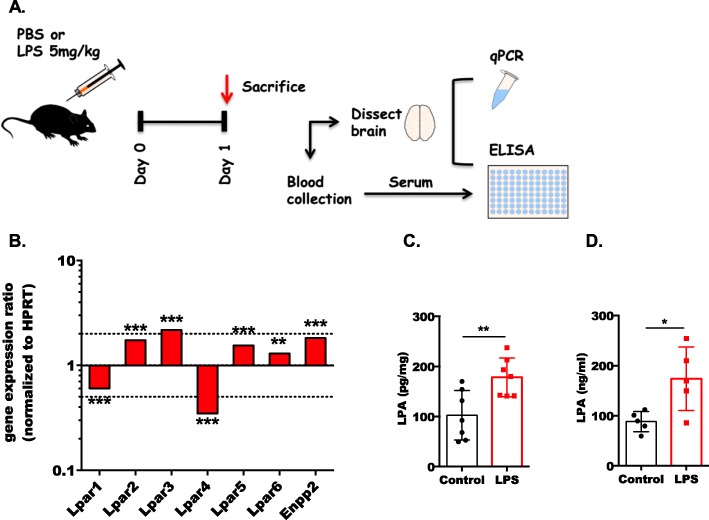
Acute inflammation differentially regulates the expression of LPARs and ENPP2 and increases LPA concentrations in mouse brain. **a** Schematic diagram of the experimental procedure. Mice (*n* = 7 per group) were injected intraperitoneally (i.p.) with PBS or LPS (5 mg/kg body weight). After 24 h, the brain and blood was collected and processed for further analyses. **b** One brain hemisphere from each mouse was processed for RNA isolation and further analysis by qPCR. Expression ratios were normalized to HPRT, and results were analyzed using the relative expression software tool (REST; **p < 0.01, ***p < 0.001; pairwise re-allocation test). The dotted lines indicate 2-fold up- or down-regulation. An LPA ELISA was utilized to quantitate LPA in the brain (*n* = 7 mice per group; (**c**)) and serum (*n* = 5 mice per group; (**d**)). Results are expressed as mean ± SD (*p < 0.05, **p < 0.01; unpaired Student’s *t* test)

**Fig. 2:**
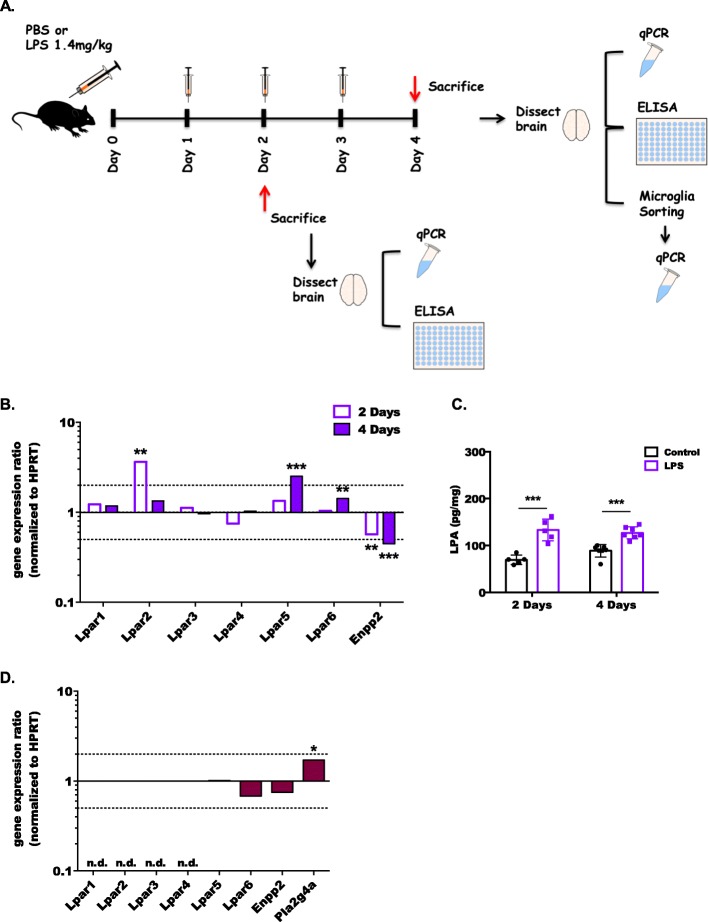
Chronic inflammation differentially regulates the expression of LPARs and ENPP2 in mouse brain and FACS-sorted microglia. **a** Schematic diagram outlining the treatment and experimental regimen. Mice (*n* = 5 per group) received i.p. injections of either PBS or LPS (1.4 mg/kg body weight) once every 24 h over a 96-h period. Dissection of the brain for further analyses was performed after 48 and 96  h. **b** One dissected hemisphere from each mouse was processed for RNA isolation and qPCR analysis. Expression ratios were normalized to HPRT and results were analyzed using the relative expression software tool (REST; **p < 0.01, ***p < 0.001; pairwise re-allocation test). The dotted lines indicate 2-fold up- or downregulation. **c** The levels of LPA in the brain (at 48 and 96 h) were quantified using an ELISA. Results are expressed as mean ± SD (***p < 0.001; unpaired Student’s *t* test). **d** In a second cohort of animals (*n* = 5), microglia were isolated from freshly dissected brains by FACS 96 h post LPS treatment. Sorted microglia were processed for RNA isolation and further analysis by qPCR. Expression ratios of LPAR1-6, ENPP2, and PLA2G4 (coding for cPLA2) were normalized to HPRT. Results were analyzed using the relative expression software tool (REST; *p < 0.05; pairwise re-allocation test).

Acute inflammation was induced via a single intraperitoneal (i.p.) injection of 5 mg/kg LPS (*n* = 7 mice). Mice were euthanized 24 h later. Chronic treatment was induced by i.p. injections of either 1.4 mg/kg LPS or PBS once every 24 h over 48 h (*n* = 5 mice per group) or 96 h (*n* = 7 mice per group) period (2 and 4 injections in total, respectively). Twenty-four hours after the last injection, animals were euthanized, perfused with ice-cold PBS, and the brains were collected for microglia isolation, RNA isolation, and LPA enzyme-linked immunosorbent assay (ELISA), as described below. Treatment regimens are outlined in Figs. 1 and 2, respectively.

### Tissue dissociation for microglia isolation

Animals (8–12 weeks) were transcardially perfused under anesthesia with ice-cold PBS, and the brains were removed and stored briefly in HBSS. The brains were homogenized in dissection buffer (HBSS containing 20 U/ml DNase) using a Dounce homogenizer; cells were strained through a 40-μm filter and pelleted by centrifugation. Cell pellets were resuspended in 3 ml of 70% Percoll, overlaid with 3 ml of 30% Percoll. Centrifugation was performed for 25 min at 800×*g* with slow acceleration and no breaks. Myelin was carefully removed, and the interphase containing microglia (2 ml) was collected in falcon tubes containing 8 ml ice-cold HBSS. The interphases from the 3 brains were pooled together in order to ensure a high number of microglia. Samples were centrifuged for 5 min at 800×*g*, the supernatants were discarded, and the cell pellets were processed for fluorescence-activated cell sorting (FACS).

### FACS of microglia

For cell sorting dissociated cell pellets were resuspended in 100 μL of PBS containing Fixable Viability Dye eFluor 780 (1:1000; Invitrogen, Waltham, MA, USA) as a viability marker and incubated for 5 min on ice. Microglia were washed with ice-cold HBSS, centrifuged for 5 min at 500×*g*, resuspended in purified rat anti-mouse CD16/CD32 (Mouse BD Fc Block™) Clone 2.4G2 (1:400; BD Biosciences) and incubated for 20 min on ice. For the final staining, the samples were incubated in 100 μl of FACS buffer (2% BSA in HBSS) including V450 rat anti-CD11b Clone M1/70 (1:400; BD Biosciences) and PerCP-Cy™5.5 rat anti-mouse CD45 Clone 30-F11 (1:400; BD Biosciences) for 25 min on ice. Samples were centrifuged for 5 min at 500×*g*, the supernatants were discarded, and the cell pellets were resuspended in 400 μl of FACS buffer. Samples were sorted on a FACSARIA Fusion II (BD Biosciences) directly into 350 μl of RLT lysis buffer (Qiagen, Hilden, Germany), immediately vortexed to ensure lysis, and placed on ice. Microglia were defined as living CD11b^+^CD45^low^ cells. FACS-sorted microglia, ranging from 70,000–150,000 cells, were stored at − 80 **°**C until RNA isolation.

### Tissue dissociation for RNA isolation or LPA ELISAs

Mice were transcardially perfused under anesthesia with ice-cold PBS, and the brains were dissected, divided in two hemispheres, and stored briefly in HBSS till the end of all the perfusions. For future RNA isolation, the hemispheres were lysed in QIAzol Lysis Reagent (Qiagen, Hilden, Germany) and stored at − 80 **°**C until further use. For quantification of LPA concentrations, each hemisphere was weighted and homogenized in PBS (100 μg tissue/ml PBS). After two freezing-thawing circles, the samples were centrifuged, and the supernatants were immediately used for LPA ELISA (Cloud-Clone, Katy, TX, USA).

### Quantitative RT-PCR (qPCR) analysis

Samples containing the sorted microglia or the brain hemispheres were thawed on ice. Total RNA was extracted using the RNeasy Micro kit and RNeasy Lipid Tissue Mini Kit respectively (Qiagen, Hilden, Germany) and quantitated using NanoDrop (Thermo Fisher Scientific, Waltham, MA, USA). RNA was reverse-transcribed using the SuperScript® III reverse transcription kit (Invitrogen, Waltham, MA, USA). qPCR was performed on an Applied Biosystems 7900HT Fast Real Time PCR System using the QuantiTect SYBR® Green PCR kit (Qiagen, Hilden, Germany). Amplification of murine hypoxanthine-guanine phosphoribosyltransferase (HPRT) was performed on all samples as internal control for variations in mRNA concentration. Gene-specific primers were purchased from Qiagen, and the primer sequences of target genes are listed in Table 1.

**Table 1:** Primers used for real time PCR analyses

Gene	Detected transcripts	Amplicon Size
*Lpar1*	NM_010336 (3362 bp) NM_172989 (3451 bp)	94
*Lpar2*	NM_020028 (5701 bp)	94
*Lpar3*	NM_022983 (2494 bp)	99
*Lpar4*	NM_175271 (4237 bp)	96
*Lpar5*	NM_001163268 (1458 bp) NM_001163269 (1393 bp) XM_355812 (1458 bp) XM_917685 (1458 bp) XM_981874 (1387 bp) XM_006506340 (5219 bp)	100
*Lpar6*	NM_175116 (2468 bp)	118
*Enpp2*	NM_001136077 (3617 bp) NM_015744 (3461 bp) NM_001285994 (3536 bp) NM_001285995 (3605 bp) XM_006520593 (3435 bp) XM_006520594 (3423 bp) XM_006520595 (3360 bp) XM_006520596 (3348 bp) XM_006520597 (3227 bp) XM_006520598 (3215 bp) XM_006520599 (2971 bp)	95
*Pla2g4a*	NM_008869 (2869 bp) XM_006529252 (2859 bp)	98

### MTT assay

Viability of PMM in the presence of the indicated inhibitors was assessed using the MTT assay. Cells plated in PDL-coated 96-well plates were treated with the indicated compounds. MTT (1.2 mM; in serum-free medium) was added to cells and incubated for 1.5 h. Cells were washed with PBS, and cell lysis was performed with isopropanol/1 M HCl (25:1; v/v) on a rotary shaker at 1200 rpm for 15 min. Absorbance was measured at 570 nm on a Victor 1420 multilabel counter (Wallac) and corrected for background absorption (650 nm).

### Immunoblotting

PMM (cultured on PDL-coated 12-well plates at a density of 4 × 10^5^ cells/well) were used for detection of COX-2, Arg1, and the phosphorylation of p65-NF-kB, c-Jun, STAT1, and STAT3. At the specific time points, treated cells were washed twice with ice-cold PBS, lysed in RIPA buffer (50 mM Tris-HCl pH 7.4, 1% NP-40, 150 mM NaCl, 1 mM Na_3_VO_4_, 1 mM NaF, 1 mM EDTA) containing protease inhibitors (Sigma; aprotinin, leupeptin, pepstatin: 1 μg/ml each), 10 μM phenylmethylsulfonyl fluoride (PMSF), and phosphatase inhibitor cocktail (Thermo Scientific, Waltham, MA, USA), scraped and centrifuged at 12,000×*g* for 10 min. Protein content was determined using BCA kit (Thermo Scientific) and BSA as standard. Protein samples (5 μg) were separated on 10% SDS-PAGE gels and transferred to polyvinylidene difluoride membranes. Membranes were blocked with 5% low-fat milk in Tris-buffered saline containing Tween 20 (TBST) for 2 h at room temperature (RT) and incubated with the primary antibodies overnight with gentle shaking at 4 °C. After removal of primary antibodies, the membranes were washed for 30 min in TBST and membranes were incubated for 2 h at RT with goat anti-rabbit (1:10,000) as secondary antibody. Following three washes with TBST for 1 h, immunoreactive bands were visualized using ECL or ECL plus reagents and detected with a chemiluminescence detection system (ChemiDoc Bio-Rad, Berkeley, CA, USA). The membranes were stripped with stripping buffer (140 μl ß-mercaptoethanol in 20 ml buffer 60 mM Tris/2% SDS, pH 6.8) under gentle shaking for 30 min at 50 °C in a water bath, washed for 1 h in TBST, blocked with 5% low-fat milk in TBST for 1 h at RT, and probed with the phospho- or pan antibodies for JNK, p38, ERK1/2, p65-NF-kB, c-Jun, STAT1, and STAT3. Anti-ß-actin antibody was used for detection of the loading control. Antibody dilutions are displayed in Table 2.

**Table 2:** Dilutions of antibodies used for immunoblotting experiments

Antibody	Dilution
phospho- p65-NF-kB	1:1000
p65-NF-kB	1:1000
phospho- c-Jun	1:500
c-Jun	1:500
phospho- STAT1	1:500
STAT1	1:1000
phospho- STAT3	1:500
STAT3	1:1000
COX-2	1:1000
Arg1	1:1000
phospho- JNK*	1:1000
JNK*	1:1000
phospho- p38*	1:1000
p38*	1:1000
phospho- ERK1/2*	1:1000
ERK1/2*	1:2000
ß-actin	1:5000
ß-tubulin*	1:2000

*Immunoblotting experiments presented in the Supplement

### Flow cytometry

Flow cytometry was performed to assess the expression of CD40, CD86, and CD206 in microglia cells. Cells were seeded in PDL-coated 24-well plates and after 24 h serum-starvation, incubated in the presence of LPA or LPA plus the antagonists for 12 and 24 h. Cells were collected; blocked using the Ultra V blocker (Thermo Scientific); incubated with PE anti-CD40 (1:50), APC anti-CD86 (1:50), or PE anti-CD206 antibody (1:100); and fixed and measured using a Guava easyCyte 8 Millipore flow cytometer.

### ELISA

The cellular supernatants were collected, snap frozen in liquid N_2_, and kept at − 80 °C until further use. IL-1β, TNFα, IL-6, CCL5 (RANTES), CXCL2 (MIP-2), and CXCL10 (IP-10) concentrations were quantitated using the murine ELISA development kits (Peprotech, NJ, USA) [26]. The assays were performed according to manufacturer’s instructions. Concentrations of cytokines and chemokines were determined using an external standard curve.

### Determination of nitric oxide (NO)

The accumulated total nitrate levels were measured in the supernatant of cells that were incubated with LPA in the absence or presence of antagonists in serum-free medium using the total nitric oxide assay kit (ENZO Life Sciences, Switzerland). This assay is based on the enzymatic conversion of nitrate to nitrite by the enzyme nitrate reductase, followed by the Griess reaction to form a colored azo-dye product. The samples were processed according to manufacturer’s protocol. A standard curve was generated in the range between 0–100 μM using nitrate as standard. The total nitrate concentration per sample was determined using external calibration.

### Determination of reactive oxygen species (ROS)

Intracellular ROS levels were measured using the ROS-ID® Total ROS Detection Kit (ENZO Life Sciences, Switzerland) according to manufacturer’s instructions with slight modifications. In brief, microglia were seeded in black clear bottom 96-well plates at a density of 5 × 10^4^ cells per well. Cells were allowed to adhere overnight and then incubated in serum-free medium, containing LPA in the absence or presence of the antagonists for indicated time periods. Thirty minutes before the end of each treatment, the cells were loaded with the ROS Detection Solution. Fluorescence intensity was measured with excitation and emission wavelengths of 485 nm and 535 nm, respectively.

### Lactate dehydrogenase assay (LDH)

LDH is a cytosolic enzyme released into the culture medium upon cell lysis or damage. The CyQUANT™ LDH Cytotoxicity Assay Kit was used to quantify cytotoxicity (Invitrogen, Waltham, MA, USA). PMM were seeded in triplicate into 12-well plates at a density of 4 × 10^5^ cells per well. Serum-starved cells were incubated in serum-free medium, containing LPA in the absence or presence of the antagonists for the indicated time periods. For each time point, the supernatants were collected and transferred to CATH.a cultures.

The CATH.a neurons were seeded in 96-well plates (1 × 10^5^ cells per well) and allowed to adhere. Following overnight serum-starvation, the cells were incubated in the presence of microglia-conditioned medium. Three wells containing only medium without cells were used as background control. As a positive control, cells were incubated with the LDH positive control solution (100% release). In order to measure maximum and spontaneous release, cells were incubated with 10% Triton X-100 and assay buffer, respectively. Cells were kept at 37 °C/5% CO_2_ for 24 h and then the plate was centrifuged at 500×*g* for 5 min. One hundred microliters of the supernatants was transferred to a new 96-well plate, and 100 μl of LDH reaction solution was added to each well. The plate was incubated at 37 °C/5% CO_2_ for 30 min under gentle shaking and the absorbance at 490 nm was measured using a plate reader.

### Statistical analysis

All experiments were performed using the appropriate number of replicates per experimental group. Statistical analyses were performed using the GraphPad Prism version 6 for Mac (GraphPad Software, Inc., San Diego, CA). Data obtained from independent measurements were analyzed by one- or two-way ANOVA followed by Bonferroni’s post hoc test. For the analysis of two experimental groups, the unpaired Student’s *t* test was used. For qPCR experiments, the expression profiles of genes and associated statistical parameters were analyzed by a pairwise re-allocation test using the relative expression software tool (REST; http://www.gene-quantification.de/rest-index.html).

## Results

In a first series of experiments, we determined gene expression levels of all LPAR subtypes and ATX (Enpp2) in total brain homogenates of C57BL/6 J mice by qPCR. Under basal conditions we detected expression of all LPARs in the order LPAR1 > 6 > 5 > 4 > 2 > 3 (Fig. S1A). We then tested whether systemic inflammation modulates LPAR and ENPP2 expression in the brain. For these experiments, C57BL/6 J mice received a single high (100 μg/20 g body weight) or four consecutive daily low (28 μg/20 g body weight) dose injections of LPS. Treatment and experimental regimens are shown in Figs. 1a and 2a.

In response to septic conditions, LPAR1 and LPAR4 were downregulated, while LPAR2, 3, 5, and 6 as well as ENPP2 were transcriptionally upregulated (Fig. 1b). In line with increased ENPP2 expression, brain LPA concentrations were also significantly elevated in response to LPS (100 vs. 190 pg/mg; PBS vs. LPS respectively; Fig. 1c). Also, serum LPA concentrations were significantly increased in response to endotoxin (88 vs 174 ng/ml; PBS vs. LPS; Fig. 1d).

Chronic inflammatory conditions revealed a slightly different regulatory pattern in the brain: At day 2 of treatment, LPAR2 was significantly upregulated, while ENPP2 expression was decreased (Fig. 2b). At day 4, LPAR5 and 6 were significantly upregulated, while ENPP2 was still downregulated. Brain LPA concentrations were increased at both time points in response to LPS treatment (71 vs. 133 and 91 vs. 128 pg/mg at day 2 and day 4, respectively; Fig. 2c). At day 4, the brains from a second cohort of PBS- or LPS-treated animals were dissociated and, FACS-sorted CD45^low^/CD11b^high^ microglia were analyzed by qPCR to get an indication about cell-specific LPAR expression. Under basal conditions, only LPAR5 and 6 were detectable in sorted microglia (Fig. S1B). In comparison to vehicle, LPAR5 remained unaffected while LPAR6 was downregulated in the chronic LPS treatment regimen (Fig. 2d). ENPP2 was also downregulated while PLA2G4A (the resulting protein generates the ATX substrate lysophosphatidylcholine) was upregulated (Fig. 2d). These findings demonstrate regulatory potential of systemic inflammation on the ATX/LPA/LPAR axis in brain and microglia.

**Fig 3:**
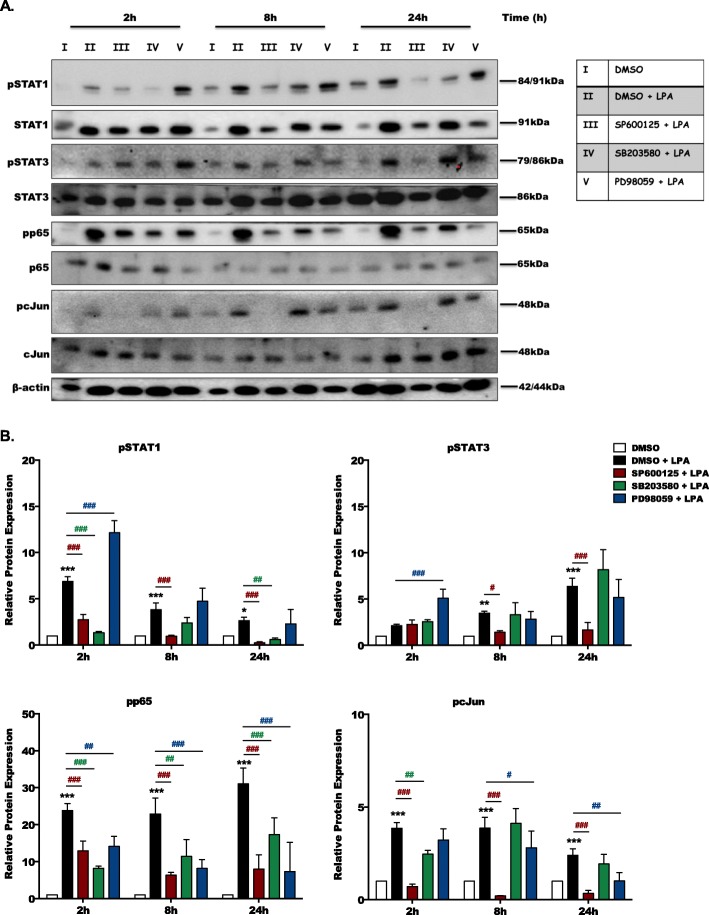
MAPK activation controls LPA-induced transcription factor phosphorylation. **a**) Primary microglia cells, isolated from C57BL/6 J pups (P0-P4), were cultured in PDL-coated 12-well plates and serum-starved overnight. Cells were incubated with DMSO (vehicle, highest final concentration 0.01%) and DMSO plus LPA (1 μM) in the absence or presence of SP600125 (10 μM), SB203580 (10 μM), or PD98059 (10 μM) for the indicated time periods. Cell protein lysates were collected and phosphorylated, and total STAT1, STAT3, p65-NF-kB, and c-Jun proteins were detected by immunoblotting. Protein/loading control ratios were normalized to the ratio of unstimulated microglia and one representative blot for each protein is presented. **b** Densitometric analysis from four independent immunoblot experiments. Results are presented as mean values + SD. (*p < 0.05, **p < 0.01, ***p < 0.001 compared to DMSO-treated cells; ^#^*p* < 0.05, ^##^*p* < 0.01, ^###^*p* < 0.001 each individual inhibitor compared to LPA-treated cells; two-way ANOVA with Bonferroni correction)

Using three different inhibitors (SP600125 for JNK1-3, SB203580 for p38, and PD98059 for MEK, an upstream kinase of ERK1/2), we studied the involvement of the different MAPK branches during LPA-mediated phosphorylation of STAT1, STAT3, p65, and c-Jun transcription factors inducing an inflammatory microglia phenotype. The inhibitors were without inadvertent effects at 10 μM (used throughout all experiments described below), however, at 20 μM compromised cell viability (Fig. S2). SP600125 and SB203580 inhibited phosphorylation of STAT1, while SP600125 specifically suppressed STAT3 phosphorylation (Fig. 3a; bar graphs in Fig. 3b show densitometric evaluation of immunoblots). All inhibitors attenuated LPA-mediated phosphorylation of p65 and c-Jun. Pathway analysis in BV-2 cells revealed inhibition of LPA-induced JNK, p38, and ERK1/2 phosphorylation by the LPA5-specific inhibitor TCLPA5 (Fig. S3). In addition to phosphorylation, LPA also mediates upregulation of total STAT1, STAT3, and p65 (but not c-Jun) protein expression in primary microglia that is inhibited most prominently by JNK antagonism (Fig. S4). LPA-mediated p65, STAT1, STAT3, and c-Jun phosphorylation in BV-2 cells was decreased to baseline levels by SP600125 treatment (Fig. S5).

**Fig. 4:**
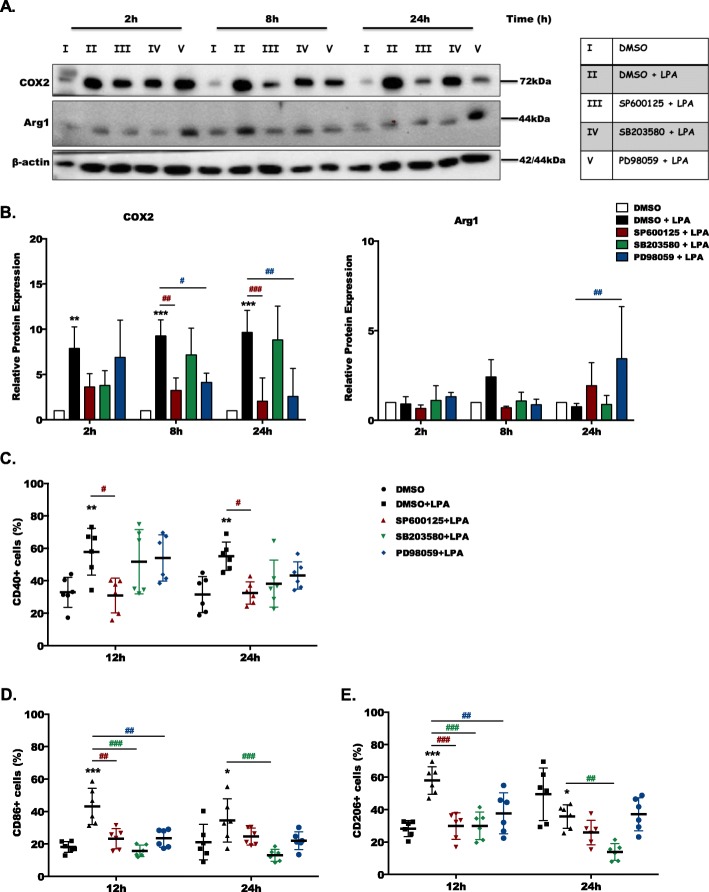
MAPK family members differentially regulate the LPA-induced pro-inflammatory phenotype of microglia. **a** Serum-starved primary microglia were treated with DMSO and DMSO plus LPA (1 μM) in the absence or presence of SP600125 (10 μM), SB203580 (10 μM), or PD98059 (10 μM) for the indicated times. Cell lysates were collected, and protein expression of COX-2 and Arg1 was monitored by immunoblotting. One representative blot for each protein and **b** the densitometric analysis (mean + SD) from four independent experiments is presented. **c**–**e** Serum-starved (overnight) primary microglia were cultivated in the presence of DMSO and DMSO plus LPA (1 μM) in the absence or presence of SP600125 (10 μM), SB203580 (10 μM), or PD98059 (10 μM) for the indicated time periods. Cells were stained with PE-conjugated anti-CD40, APC-conjugated anti-CD86, or PE-conjugated anti-CD206 antibodies and analyzed using a Guava easyCyte 8 Millipore flow cytometer. Results from three individual experiments performed in duplicates are shown as mean values ± SD. (*p < 0.05, **p < 0.01, ***p < 0.001 compared to DMSO-treated cells; ^#^*p* < 0.05, ^##^*p* < 0.01, ^###^*p* < 0.001 each individual inhibitor compared to LPA-treated cells; two-way ANOVA with Bonferroni correction)

In the next set of experiments we analyzed the impact of MAPK antagonism on the expression of pro- (COX-2, CD40, and CD86) and anti-inflammatory (Arg1 and CD206) markers using immunoblotting and flow cytometry. Immunoblotting experiments revealed that LPA induced COX-2 expression at 2, 8, and 24 h between 8 and 10-fold (Fig. 4a, b, left panel). Inhibition of JNK and ERK1/2 decreased COX-2 signals back to baseline levels. Arg1 was upregulated by LPA at 8 h, and ERK inhibition significantly upregulated Arg1 signal intensities at 24 h (Fig. 4b, right panel). LPA treatment significantly upregulated the percentage of CD40-, CD86-, and CD206-positive cells at 12 and 24 h of treatment, which are used as membrane marker proteins in the disputed [28] M1/M2 microglia paradigm (Fig. 4c–e). CD40 expression was downregulated specifically by SP600125 (Fig. 4c). Upregulation of CD86 and CD206 by LPA was attenuated in response to all MAPK inhibitors after 12 h, while after 24 h, only SB203580 suppressed upregulation (Fig. 4d and e). In terms of LPA-mediated upregulation of COX-2, Arg1 and membrane marker proteins comparable results were obtained in BV-2 cells (Fig. S6). In this microglia cell line, SP600125 decreased COX-2, CD40, and CD86 and increased Arg1 expression (Fig. S6).

**Fig. 5:**
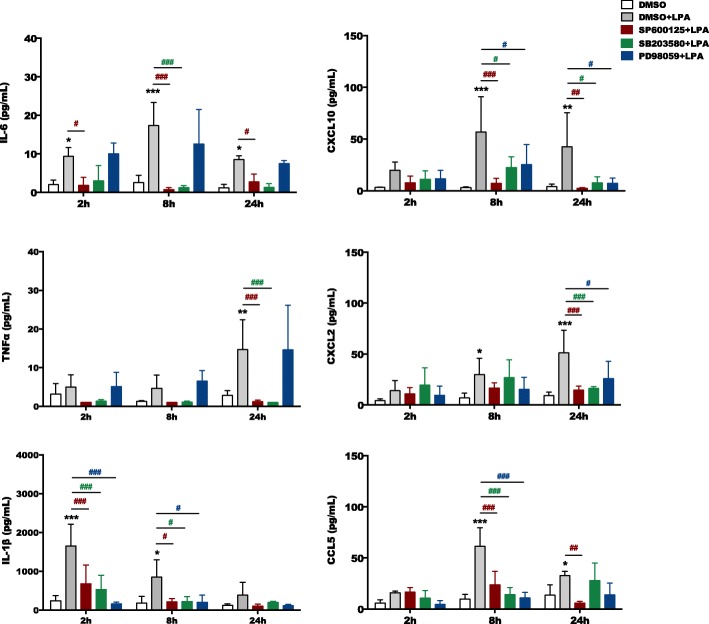
MAPK signaling directs LPA-mediated secretion of pro-inflammatory cytokines and chemokines. Primary microglia were cultured on PDL-coated 12-well plates and serum-starved overnight. The supernatants were collected after incubation with DMSO and DMSO plus LPA (1 μM) in the absence or presence of SP600125 (10 μM), SB203580 (10 μM), or PD98059 (10 μM) for the indicated time periods. ELISA was used to quantitate the concentrations of IL-6, TNFα, IL-1β, CXCL10 (IP-10), CXCL2 (MIP-2), and CCL5 (RANTES). Results shown represent the mean + SD from four independent experiments (*p < 0.05, **p < 0.01, ***p < 0.001 compared to vehicle control; ^#^*p* < 0.05, ^##^*p* < 0.01^###^, *p* < 0.001 each inhibitor compared to LPA treated cells; two-way ANOVA with Bonferroni correction)

Next, we quantitated cytokine (IL-6, TNFα, and IL-1ß) and chemokine (CXCL10, CXCL2, and CCL5) concentrations in the supernatant of vehicle- and LPA-treated primary PMM in the absence or presence of MAPK inhibitors (Fig. 5). ELISA analyses revealed that LPA induced the secretion of all cyto-/chemokines analyzed during these experiments. Of these, IL-1ß showed an immediate (maximum at 2 h), IL-6, CXCL10, and CCL5 a medium (maximum at 8 h) and TNFα and CXCL2 a late (maximum at 24 h) response towards LPA treatment. SP600125 and SB203580 decreased IL-6 and TNFα secretion to baseline levels, while all MAPK inhibitors suppressed secretion of IL-1ß, CXCL10, CXCL2, and CCL5 (Fig. 5). In BV-2 cells, qualitatively similar observations were made for LPA-stimulation and the SP600125 inhibitor (Fig. S7).

**Fig. 6:**
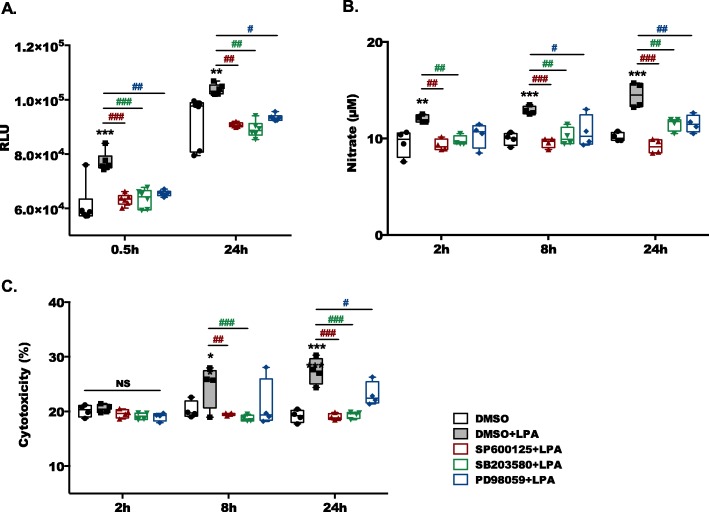
Inhibition of MAPK pathways abrogates LPA-induced ROS and NO production and decreases microglial neurotoxicity. **a** A ROS assay kit was used to determine changes in the ROS levels generated by primary murine microglia. Serum-starved cells were treated with DMSO and DMSO plus LPA (1 μM) in the absence or presence of SP600125 (10 μM), SB203580 (10 μM), or PD98059 (10 μM) for 30 min and 24 h, followed by 30 min incubation with carboxy-H_2_DCFDA, and the fluorescence intensity was evaluated. Results from three experiments are presented as mean values ± SD. RLU, relative luminsescence units. **b** Serum-starved microglia cells were incubated with vehicle DMSO and DMSO plus LPA (1 μM) in the absence or presence of SP600125 (10 μM), SB203580 (10 μM), or PD98059 (10 μM) for the indicated time periods, and the production of NO was determined by measuring the total nitrate concentration in the supernatants. Data (4 separate experiments) are presented as mean values ± SD. **c** CATH.a neurons were incubated for 24 h with conditioned media collected from LPA-treated primary microglia in the absence or presence of SP600125 (10 μM), SB203580 (10 μM) or PD98059 (10 μM) for the indicated time periods. The LDH levels were detected and cytotoxicity was calculated according to the manufacturer’s recommendations. (*p < 0.05, **p < 0.01, ***p < 0.001 compared to vehicle control; ^#^*p* < 0.05, ^##^*p* < 0.01^###^_,_*p* < 0.001 each inhibitor compared to LPA treated cells; two-way ANOVA with Bonferroni correction)

In the last set of experiments, we analyzed the effects of LPA (absence or presence of MAPK inhibitors) on ROS and NO formation, which are potent mediators of microglia neurotoxicity and explored the impact of MAPK inhibition on neurotoxic properties of PMM-conditioned medium on CATH.a neurons. LPA increased ROS production at 0.5 and 24 h (Fig. 6a) and this was decreased to baseline levels by all three inhibitors. A comparable pattern was obtained for NO production where the LPA-mediated increase was attenuated in response to MAPK pathway inhibition (Fig. 6b). Finally, we determined potential neurotoxic properties of PMM-conditioned media. For these experiments, PMM were incubated in the presence of vehicle or LPA in the absence and presence of MAPK inhibitors. PMM supernatants were collected after 2, 8, and 24 h and transferred to murine CATH.a neurons. Neuronal cell death was quantified using an LDH activity kit. To exclude direct effects of MAPK inhibitors on CATH.a LDH release, cells were incubated in the presence of conditioned media collected from DMSO+LPA-treated PMM and subsequently added antagonists. In these control experiments, no inhibitor-mediated effects on LDH release were observed (Fig. S8). Medium collected from LPA-stimulated PMM significantly increased neuronal LDH release 8 and 24 h post addition of PMM-conditioned medium. Inhibition of JNK and p38 signaling blunted these effects back to baseline levels while ERK1/2 inhibition was less effective (Fig. 6c). In BV-2 cells, the effects were quantitatively less pronounced but qualitatively reflected findings obtained with PMM (Fig. S9).

## Discussion

**Fig. 7:**
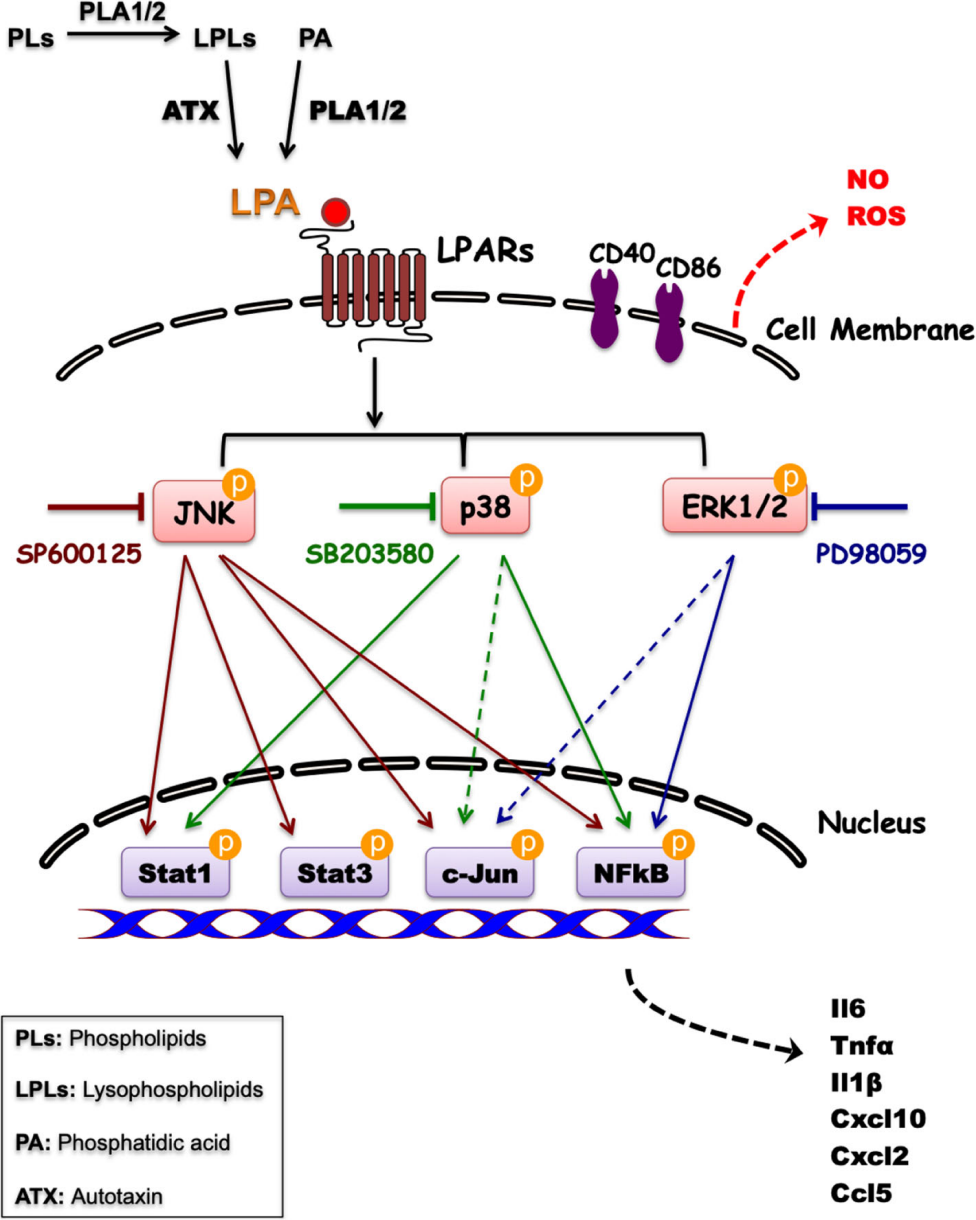
Graphical summary of results. Increased LPA levels generated via the ATX or cPLA2 pathways promote a distinct pro-inflammatory phenotype in microglia via LPAR signal transmission. Interference with the three MAPK signaling pathways regulated the LPA-induced inflammatory state in different ways. The individual steps between LPARs and MAPKs are not presented in the graph since these parts of the pathways were not experimentally addressed during the present study

Neuroinflammation is considered a critical and sometimes contributing factor during neurodegeneration. During the present study, we have shown that LPA treatment of PMM induces proinflammatory signaling events that are executed by MAPK cascades. Findings of the present study are graphically summarized in Fig. 7.

In inflammatory disease models, LPA can exert protective or harmful effects depending on whether acute or chronic experimental models are studied [29, 30]. A functional ATX/LPA/LPAR-signaling axis is indispensable for normal CNS development and modulates cellular functions of neurons, oligodendrocytes, astrocytes, and microglia [31]. Depending on context, LPA can induce protective or neurotoxic microglia polarization with the signaling output most likely depending on their state of pre-activation. Experimental animal models and clinical data support the notion that LPA can be protective in inflammatory diseases: In the murine experimental autoimmune encephalomyelitis model of multiple sclerosis (MS) and in MS patients, concentrations of several LPA species are reduced, and subsequent deficiency in LPAR2 signaling in immune cells promotes disease progression [32].

On the other hand, there is evidence that pharmacological inhibition of LPA synthesis or downstream signaling has beneficial effects in rodent models of neuropathic pain [33], demyelination [34], traumatic brain injury [35], and the experimental autoimmune encephalomyelitis model [36]. These preclinical models present a neuroinflammatory component that is (at least partially) mediated by LPA-activated microglia. Since any biological response to increased LPA levels presumably depends on abundance and binding capacity of the different LPARs, we first studied the impact of acute and chronic LPS application on receptor gene expression in the brain and FACS-sorted microglia. These analyses revealed expression of LPAR1 > 6 > 5 > 4 > 2 > 3 in brains of adult C57BL/6 J mice under basal conditions (Fig. S1A). In line with a strict spatiotemporal expression profile of LPARs, Suckau and colleagues reported that only LPAR1, LPAR2_,_ LPAR4_,_ and LPAR6 (but not LPAR3 and LPAR5) transcripts were detected in C57BL/6 J brain from embryonic day 16 until postnatal day 30 [37]. Kwon and colleagues determined LPAR expression in the brain of adult ICR (CD-1) albino mice and reported an expression pattern of LPAR1 > LPAR4 > LPAR6 > LPAR2 ≥ LPAR3 > LPAR5 [38]. These findings indicate that LPAR expression is not only under developmental regulation but also differ between different mouse strains. In FACS-sorted microglia, we could only detect LPAR5 and LPAR6 (Fig. S1B). This finding is consistent with the fact that LPAR5 and 6 are the major isoforms expressed by primary murine microglia isolated from C57BL/6 J mice [26].

Both cPLA2 and ATX are subject to upregulation by inflammatory stimuli, in particular TNFα [39, 40]. During the present study, we obtained evidence that LPS-induced systemic inflammation affects gene expression of cPLA2, ATX, and LPA concentrations in the brain (Figs. 1 and 2). These findings indicate that enzymes involved in brain LPA synthesis are regulated by systemic inflammation. Accordingly, acute and chronic inflammatory conditions were accompanied by a 2-fold increase in brain LPA levels. In response to traumatic brain injury, LPA levels are elevated in CSF of patients (0.05 controls vs. 0.27 μM post injury) and mice exposed to controlled cortical impact (CCI) traumatic brain injury (0.8 and 2 μM, prior vs. post injury) [35]. This study revealed that a single-dose administration of anti-LPA monoclonal antibodies mitigates edema, hemorrhage, and long-term behavioral deficits in the murine CCI model. In light of upregulated LPAR3 expression in the high dose LPS treatment regimen (Fig. 1), it is of importance that exogenous LPA can fuel endogenous LPA production via this LPAR subtype [41]. During spinal cord injury, parenchymal LPA concentrations increase from 75 to 725 pmol/mg protein (naïve vs. 3 d post injury) and contribute to secondary injury manifested as demyelination [34]. In a series of seminal papers, the Ueda group demonstrated that increased LPA synthesis and LPAR-mediated demyelination is the committing step during induction of neuropathic pain [33, 42].

During an earlier study, we could show that LPA provides an inflammatory “go” signal via LPAR5-mediated signaling in primary murine microglia [27]. Here, we confirmed LPAR5 involvement in the BV-2 cell line using the TCLPA5 inhibitor (Fig. S3). LPAR5 engagement activates PKD2 [27], a member of the PKD family, that is coupled to transactivation of the MAPK pathways [43]. To address a functional role of MAPK cascades (JNK, p38, ERK1/2) downstream of LPAR5/PKD2 during microglia polarization, we used specific antagonists to interfere with either signaling branch known to activate inflammatory transcription factors. Analysis of MAPK-dependent transcription factor phosphorylation (Fig. 3) revealed that LPA induces activation of STAT3 via JNK, STAT1 via JNK, and p38, p65 by all MAPK members, and c-Jun by JNK and ERK1/2. Although all of these transcription factors are implicated in neuroinflammation, STAT3 can take pro- as well as anti-inflammatory properties in microglia [44, 45]. On the other hand, STAT1 usually promotes inflammation, cytokine secretion, and production of NO and ROS [46] while NF-κB-mediated microglia activation results in the release of pro-inflammatory cytokines and neurotoxicity [47].

Activating signals that diverge from MAPK pathways are critical mediators of neuroinflammation and play important roles in neurodegenerative diseases [48]. In line, activation of MAPK pathways drives COX-2 and iNOS expression and contributes to microglia-induced neuronal cell death [49]. ROS, together with NO, can give rise to the formation of peroxynitrite, a reactive species able to induce neuronal apoptosis [50]. In the present study, inhibition of JNK and ERK1/2 reduced LPA-induced COX-2 expression back to baseline levels (Fig. 4) while all MAPK inhibitors effectively suppressed ROS and NO production (Fig. 6). The p38 MAPK axis regulates microglial cytokine synthesis and secretion in response to diverse stressors [51] and relieves the inhibitory control of autophagy on inflammation in LPS-activated microglia [52]. Data of the present study show that pharmacological antagonism of the MAPK pathways suppresses cytokine synthesis/secretion in LPA-activated primary microglia (Fig. 5).

Several strategies to reprogram microglia towards a beneficial phenotype were proposed [53] and the ATX/LPA/LPAR axis might offer new pharmacological opportunities for repolarization. Along the ATX/LPA/LPAR axis, several antagonists are in clinical trials. NCT03830125, a first-in-human study of BBT-877, is recruiting to assess the safety and tolerability of this ATX inhibitor. A phase 2a study with GLPG1690 (also an ATX inhibitor) was completed and confirmed target engagement [54]. LPAR antagonists entered clinical trials to test their pharmacological efficacy (e.g., the LPAR1 inhibitors BMS-986020 and SAR100842; NCT01766817 and NCT01651143, respectively). AS2717638, an LPAR5 inhibitor, displayed potent analgesic effects against neuropathic and inflammatory pain in preclinical rodent models [55], conditions mediated by activated microglia [56]. We have demonstrated that inhibition of LPAR5 by the inhibitor TCLPA5 reduces secretion of inflammatory cyto-/chemokines from primary microglia and reduces p38 and ERK1/2 activation [27]. Recently, we could show that the LPAR5 antagonists AS2717638 (a piperidine-based compound) and Compound3 (Cpd3; a diphenylpyrazole derivative) significantly dampen the inflammatory response of BV-2 cells towards LPA [57]. MAPK signaling regulates several functions of the immune system and an understanding of these pathways might be critical to develop strategies to reprogram microglia in neurological disorders. Neuropathic pain models that are associated with persistent LPA signaling and subsequent activation of MAPK cascades might be candidates for such an approach [42]. Indeed, a series of recent reports demonstrated that pharmacological intervention strategies that attenuate MAPK signaling pathways (either directly or indirectly) alleviate hyperalgesia in preclinical animal models of neuropathic pain [58–61]. In one of our earlier proteome studies, we have shown that LPA affects the abundance of a number of metabolic enzymes involved in glycolysis [20]. Therefore pharmacological modulation of metabolic pathway utilization within the concept of immunometabolism [62] might offer novel and effective approaches to reprogram potentially neurotoxic microglia phenotypes [53].

Based on the present results (graphically summarized in Fig. 7), we propose that MAPK antagonism might provide an efficient point of action to pharmacologically shift LPA-induced microglia polarization.

## Conclusions

We conclude that our in vivo data obtained in C57BL/6 J mice demonstrate a clear connection between systemic inflammation and modulation of the ATX/LPA/LPAR axis in the brain. Our in vitro studies provide further functional support for a central role of MAPK signaling during LPA-induced microglia polarization towards a potentially neurotoxic phenotype.

## Supplementary information

**Additional file 1.** Supplementary Information.

## Data Availability

All data generated or analyzed during this study are included in this published article and its supplementary information.

## Abbreviations

APC: Allophycocyanin
Arg1: Arginase-1
ATX: Autotaxin (gene name: ENPP2)
c-Jun: Transcription factor AP-1 (Proto-oncogene c-Jun)
CCL5: C-C motif chemokine 5
CD206: Macrophage mannose receptor 1
CD40: Tumor necrosis factor receptor superfamily member 5
CD86: T-lymphocyte activation antigen CD86
CNS: Central nervous system
COX-2: Prostaglandin G/H synthase 2 (cyclooxygenase-2)
cPLA2: Cytosolic phospholipase A2 (gene name: PLA2G4A)
CSF: Cerebrospinal fluid
CXCL10: C-X-C motif chemokine 10
CXCL2: C-X-C motif chemokine 2
Cy: Cyanine
DAG: Diacylglycerol
DGK: Diacylglycerol kinase
DMEM: Dulbecco’s modified Eagle’s medium
DMSO: Dimethylsulfoxide
ELISA: Enzyme-linked immunosorbent assay
ENPP2: Ectonucleotide pyrophosphatase/phosphodiesterase family member 2
ERK1/2: Extracellular signal-regulated kinase 1/2
FACS: Fluorescence-activated cell sorting
FCS: Fetal calf serum
GPCR: G protein-coupled receptor
HBSS: Hank’s balanced salt solution
IL-1ß: Interleukin-1 beta
HPRT: Hypoxanthine-guanine phosphoribosyltransferase
IL-6: Interleukin-6
iNOS: Inducible nitric oxide synthase
JNK: c-Jun N-terminal kinase 1
LDH: Lactate dehydrogenase
LPA: Lysophosphatidic acid
LPAR1-6: LPA receptor 1-6
LPS: Lipopolysaccharide
MAPK: Mitogen-activated protein kinase
MEK: Mitogen-activated protein kinase kinase
MS: Multiple sclerosis
MTT: 3-(4,5-dimethylthiazol-2-yl)-2,5-diphenyltetrazolium bromide
NF-κB: Nuclear factor NF-kappa-B
NO: Nitric oxide
p65: Transcription factor p65 (Nuclear factor NF-kappa-B p65 subunit)
PBS: Phosphate-buffered saline
PD98059: MEK inhibitor
PDL: Poly-D-lysine
PE: Phycoerythrin
Per-CP: Peridinin chlorophyll protein complex
PKD2: Protein kinase D family member 2
PL: Phospholipid
PMM: Primary murine microglia
qPCR: Real-time polymerase chain reaction
REST: Relative expression software tool
ROS: Reactive oxygen species
RT: Room temperature
SB203580: p38 inhibitor
SP600125: JNK1-3 inhibitor
sPLA2: Secretory phospholipase A2
STAT: Signal transducer and activator of transcription
TBST: Tris-buffered saline containing Tween20
TCLPA5: LPAR5 antagonist
TNFα: Tumor necrosis factor α
