# Supplementary material for: MAPK signaling determines lysophosphatidic acid (LPA)-induced inflammation in microglia
Source: J Neuroinflammation. 2020 Apr 23;17:127. doi: 10.1186/s12974-020-01809-1 (PMC7178949; doi:10.1186/s12974-020-01809-1)
Supplement: Supplementary file 1 — Additional file 1. Supplementary Information. [file 12974_2020_1809_MOESM1_ESM.docx]

**Supplementary Information**

**Title:** MAPK signaling determines lysophosphatidic acid (LPA)-induced inflammation in microglia

**Authors:** I. Plastira^1^, E. Bernhart^1^, L. Joshi^1^, C. Koyani^1,2^, H. Strohmaier^3^, H. Reicher^1^, E. Malle^1^, W. Sattler^1,4 *^

**Affiliations:**

^1^ Division of Molecular Biology and Biochemistry, Gottfried Schatz Research Center, Medical University of Graz, Austria.

^2^ Division of Cardiology, Department of Internal Medicine, Medical University of Graz, Austria.

^3^ Center for Medical Research, Medical University of Graz, Austria.

^4^ Center for Explorative Lipidomics, BioTechMed-Graz, Austria.

*To whom correspondence should be addressed:

Wolfgang Sattler, Division of Molecular Biology and Biochemistry, Gottfried Schatz Research Center, Medical University of Graz, Neue Stiftingtalstrasse 6/6, 8010 Graz, Austria.

Ph: +43-316-385-71950

Fax: +43-316-385-79615

email: [wolfgang.sattler@medunigraz.at](mailto:wolfgang.sattler@medunigraz.at)

**Email coauthors:**

[ioanna.plastira@medunigraz.at](mailto:ioanna.plastira@medunigraz.at); [eva.bernhart@medunigraz.at](mailto:eva.bernhart@medunigraz.at); lisha.joshi@medunigraz.at; chintan.koyani@medunigraz.at; heimo.strohmaier@medunigraz.at; helga.reicher@medunigraz.at; ernst.malle@medunigraz.at.

**A.**

**B.**

**Fig. S1. LPA receptor (LPAR1-6) expression in whole mouse brain and FACS-sorted primary microglia**

(**A**) Gene expression was monitored by qPCR and normalized to the housekeeping gene HPRT in the brain tissue of control mice. Relative expression ratios were normalized to LPAR2 (set as 1) and results were analyzed using the relative expression software tool (REST; pairwise re-allocation test; **p<0.01, ***p<0.001 vs. LPAR2).

(**B**) LPAR expression of sorted microglia from control mice. Relative expression ratios were normalized to LPAR5 (set as 1) and results were analyzed using REST (pairwise re-allocation test; **p<0.01 vs. LPAR5). n.d. = not detectable.

**Fig. S2. Evaluation of cytotoxicity of the three MAPK inhibitors in primary microglia**

The level of cytotoxicity was assessed using the MTT assay. Primary murine microglia cells were cultured in 48-well plates, serum-starved overnight (overnight) and incubated with 10 or 20 µM with the indicated inhibitors for 24 h. DMSO was used as vehicle control. Results from 2 independent experiments in triplicates are presented as mean + SD (^##^p<0.01, ^###^p<0.001 compared to vehicle control; one-way ANOVA with Bonferroni correction. NS = non significant). RLU = relative fluorescence units.


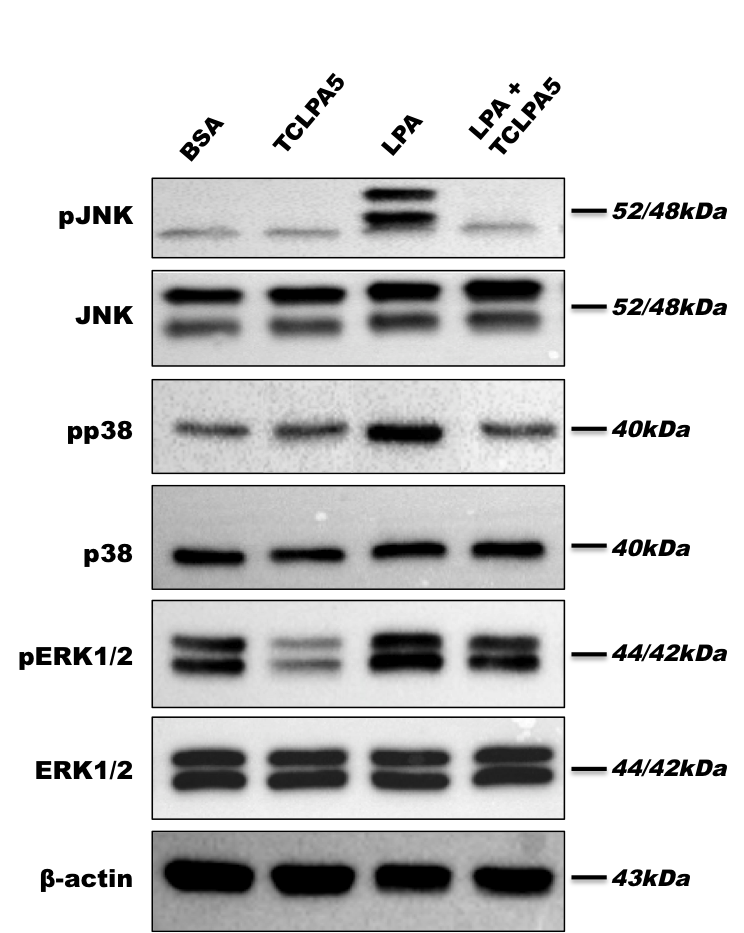


**A.**

**B.**

**Fig. S3. LPA induces MAPK activation in BV-2 cells in an LPAR5-dependent manner**

Cells, serum-starved overnight and treated with LPA (1 µM) or LPA (1 µM) in the presence of the LPAR5 antagonist TCLPA5 (5 µM) for 30 min. Cells incubated with 0.1 % BSA or TCLPA5 (5 µM) were used as negative controls. Phospho- and total JNK, p38, and ERK1/2 were detected by immunoblotting. One representative blot is shown. (**B**) Densitometric analysis of immunoblots (N=3). Results are presented as mean values + SEM (**p<0.01, ***p<0.001 compared to control; ^##^p<0.01, ^###^p<0.001 LPA plus TCLPA5 versus LPA; one-way ANOVA with Bonferroni correction).

**Fig. S4. Quantification of total STAT1, STAT3, p65-NF-kB, and c-Jun expression in primary microglia**

Densitometric analysis of the total levels of STAT1, STAT3, p65-NF-kB, and c-Jun. Protein/loading control ratios were normalized to the ratio of unstimulated cells. Results from 4 independent experiments are presented as mean values + SD. (**p<0.01; ***p<0.001 compared to DMSO-treated cells; ^#^p<0.05; ^##^p<0.01 each inhibitor compared to LPA-treated cells; two-way ANOVA with Bonferroni correction).

**A.**

**B.**


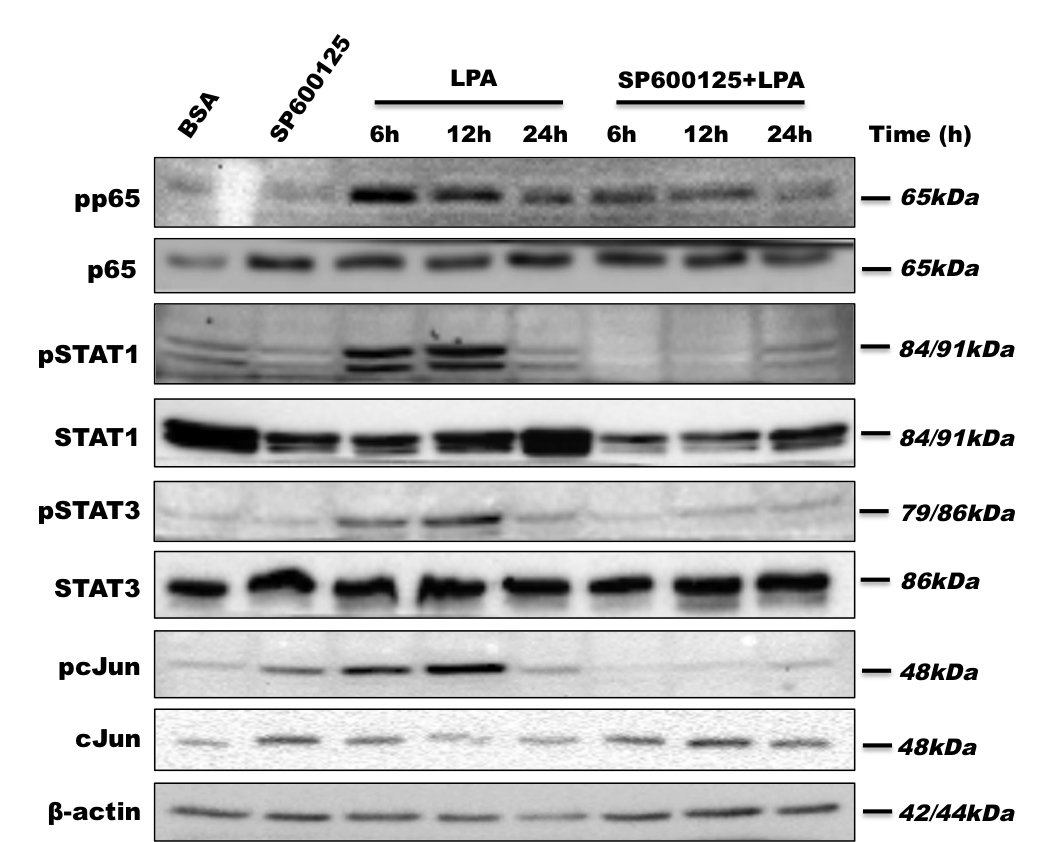


**Fig. S5. LPA-induced phosphorylation of pro-inflammatory transcription factors in BV-2 cells is under JNK control**

(**A**) Cells were serum-starved overnight and treated with LPA (1 µM) or LPA (1 µM) in the presence of SP600125 (10 µM) for the indicated time periods. Cells incubated only with 0.1 % BSA or SP600125 (10 µM) were used as negative control. Phospho- and total p65-NF-κB, STAT1, STAT3, and c-Jun were detected by immunoblotting. One representative blot is shown. (**B**) Densitometric analysis of immunoblots (N=3). Results are presented as mean values + SEM (*p<0.05; **p<0.01, ***p<0.001 compared to control; ^#^p<0.05; ^##^p<0.01, ^###^p<0.001 LPA plus SP600125 versus LPA; one-way ANOVA with Bonferroni correction).

**A.**

**B.**


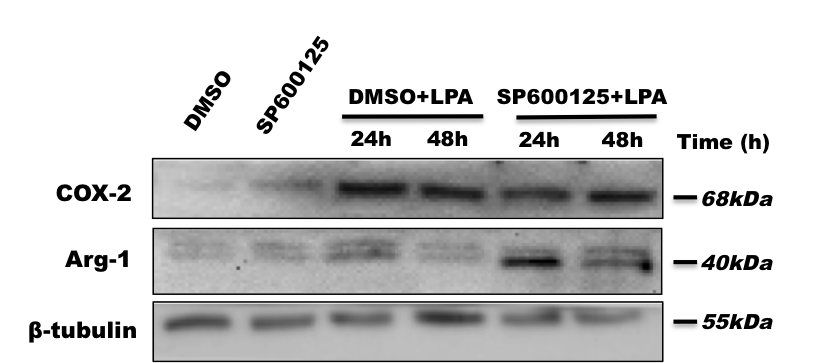


**Fig. S6. Inhibition of JNK alters polarization marker expression in BV-2 cells**

(**A**) Serum-starved (overnight) BV-2 cells were treated with DMSO and DMSO plus LPA (1 µM) in the absence or presence of SP600125 (10 µM) for 24 and 48 h. Cell lysates were collected and the expression of COX-2 and Arg-1 was monitored by immunoblotting. One representative blot and the densitometric analysis (mean + SD) from three independent experiments is presented.

(**B**) In a parallel experiment, serum-starved (overnight) BV-2 cells were cultivated in the presence of DMSO and DMSO plus LPA (1 µM) in the absence or presence of SP600125 (10 µM) for the indicated time periods. Cells were stained with PE-conjugated anti-CD40, APC-conjugated anti-CD86 or PE-conjugated anti-CD206 antibodies and analyzed using a Guava easyCyte 8 Millipore flow cytometer. Results from three individual experiments are shown as mean values + SD.

(*p<0.05; **p<0.01; ***p<0.001 compared to DMSO-treated cells; ^#^p<0.05; ^##^p<0.01 each inhibitor compared to LPA-treated cells; two-way ANOVA with Bonferroni correction).

**Fig. S7. Inhibition of JNK mitigates secretion of pro-inflammatory cytokines and chemokines by BV-2 cells**

Cells were cultured on 12-well plates and serum-starved overnight. The supernatants were collected after incubation with vehicle control (DMSO) and DMSO plus LPA (1 µM) in the absence or presence of SP600125 (10 µM) for the indicated time periods. ELISA was used to quantitate the concentrations of IL-6, TNFα, IL-1β, CXCL10 (IP-10), CXCL2 (MIP-2), and CCL5 (RANTES). Results are presented as mean + SD from three independent experiments (*p<0.05; **p<0.01; ***p<0.001 compared to vehicle control; ^#^p<0.05, ^##^p<0.01; each inhibitor compared to LPA treated cells; one-way ANOVA with Bonferroni correction).

**Fig. S8. Effect of MAPK inhibitors on neuronal death induced by primary microglia-conditioned media**

CATH.a neurons were incubated for 24 h with conditioned media collected from DMSO or DMSO+LPA-treated primary microglia cells (2, 8, and 24 h). In order to test whether MAPK inhibitors are acting on neurons, CATH.a cells were also incubated for 24 h with conditioned media from DMSO+LPA-treated microglia cells (for 2, 8, and 24 h) in the presence of each inhibitor (10 µM). The LDH levels were detected and cytotoxicity was calculated according to the manufacturer’s directions. (**p<0.01 compared to vehicle control; one-way ANOVA with Bonferroni correction).

**A.**

**B.**

**C.**

**Fig. S9. Inhibition of JNK abrogates LPA-induced ROS/NO production in BV-2 cells and decreases cytotoxicity of BV-2-conditioned media**

(**A**) Intracellular ROS levels were measured using a commercially available kit. Serum-starved (overnight) BV-2 cells were treated with DMSO, DMSO plus LPA (1 µM), and LPA in the presence of SP600125 (10 µM) for the indicated time periods, followed by 30 min incubation with carboxy-H_2_DCFDA and subsequent fluorescence intensity measurement. Results from three experiments are presented as mean values + SD.

(**B**) Serum-starved (overnight) BV-2 cells were incubated with DMSO, DMSO plus LPA (1 µM) or LPA plus SP600125 (10 µM) for the indicated time periods and the production of NO was determined by measuring the total nitrate concentration in the supernatants. Data (3 separate experiments) are presented as mean values + SD.

(**C**) CATH.a neurons were incubated for 24 h with conditioned media collected from LPA-treated BV-2 cells in the absence or presence of SP600125 (10 µM) for 24 h. The LDH levels were detected and cytotoxicity was calculated according to the manufacturer’s directions. (**p<0.01; ***p<0.001 compared to vehicle control; ^#^p<0.05, ^##^p<0.01SP600125 compared to LPA-treated cells; one-way ANOVA with Bonferroni correction).
